# Leafamine^®^, a Free Amino Acid-Rich Biostimulant, Promotes Growth Performance of Deficit-Irrigated Lettuce

**DOI:** 10.3390/ijms23137338

**Published:** 2022-06-30

**Authors:** Marthe Malécange, Maria-Dolores Pérez-Garcia, Sylvie Citerne, Renaud Sergheraert, Julie Lalande, Béatrice Teulat, Emmanuelle Mounier, Soulaiman Sakr, Jérémy Lothier

**Affiliations:** 1 Univ Angers, Institut Agro, INRAE, IRHS, SFR QUASAV, 49000 Angers, France; marthe.malecange@etud.univ-angers.fr (M.M.); maria-dolores.perez-garcia@agrocampus-ouest.fr (M.-D.P.-G.); julie.lalande@inrae.fr (J.L.); beatrice.teulat@agrocampus-ouest.fr (B.T.); jeremy.lothier@univ-angers.fr (J.L.); 2 BCF Life Sciences, Boisel, 56140 Pleucadeuc, France; rsergheraert@bcf-lifesciences.com (R.S.); emounier@bcf-lifesciences.com (E.M.); 3 Université Paris-Saclay, INRAE, AgroParisTech, Institut Jean-Pierre Bourgin (IJPB), 78000 Versailles, France; sylvie.citerne@inrae.fr

**Keywords:** *Lactuca sativa* L., biostimulant, protein hydrolysate, free amino acids, water deficit, polyamines, osmoprotectants

## Abstract

Water deficit causes substantial yield losses that climate change is going to make even more problematic. Sustainable agricultural practices are increasingly developed to improve plant tolerance to abiotic stresses. One innovative solution amongst others is the integration of plant biostimulants in agriculture. In this work, we investigate for the first time the effects of the biostimulant –Leafamine^®^–a protein hydrolysate on greenhouse lettuce *(Lactuca sativa* L.) grown under well-watered and water-deficit conditions. We examined the physiological and metabolomic water deficit responses of lettuce treated with Leafamine^®^ (0.585 g/pot) or not. Root application of Leafamine^®^ increased the shoot fresh biomass of both well-watered (+40%) and deficit-irrigated (+20%) lettuce plants because the projected leaf area increased. Our results also indicate that Leafamine^®^ application could adjust the nitrogen metabolism by enhancing the total nitrogen content, amino acid (proline) contents and the total protein level in lettuce leaves, irrespective of the water condition. Osmolytes such as soluble sugars and polyols, also increased in Leafamine^®^-treated lettuce. Our findings suggest that the protective effect of Leafamine is a widespread change in plant metabolism and could involve ABA, putrescine and raffinose.

## 1. Introduction

Substantial evidence shows that climate change is leading to a greater intensity of extreme climate events, including severe drought periods [1,2]. Water deficiency is considered one of the most important environmental factors that impedes plant growth and development, causing significant yield losses. Research efforts are focused on the development of strategies to mitigate its effects on crop yield and much attention has been paid to the use of environment-friendly strategies relying on the integration of plant biostimulants in cultivation systems.

During a water shortage, plants can establish molecular and biochemical responses [3,4] that cause morphological and physiological changes in fine. Although the understanding of the signaling network underlying these changes is still in progress [5,6], plant responses to water deficit are well characterized. They include morphological changes, mainly marked by a reduction of the leaf area and biomass [4].

These changes in plant development could be explained by changes in photosynthesis efficiency and carbon allocation [7,8], which are determinant for growth. Phytohormones are also implied in regulating physiological responses to water deficit [9,10], with a central role of ABA (Abscisic acid) through its effect on stomatal closure and the induction of protective molecules and proteins [11].

To prevent water loss and degradations during cellular dehydration, one predominant well-known response is the accumulation of osmoprotectants, i.e., small molecules non-toxic at molar concentrations that stabilize cell membranes and proteins against the denaturing effects of stress on the cellular functions [12,13]. Osmoprotectants include amino acids, e.g., proline, glutamate and γ-aminobutyric acid (GABA); carbohydrates, e.g., sucrose, hexoses and fructans; quaternary ammonium compounds, e.g., glycine betaine; and polyols, e.g., inositol and mannitol. They play a key role in maintaining the turgor pressure of plant cells by adjusting their osmotic potential [3,14]. Furthermore, osmoprotectants contribute to the reduction of reactive oxygens species (ROS) generated during abiotic stresses [15,16] by stimulating the activity of antioxidant enzymes (peroxidase, superoxide dismutase, ascorbate peroxidase and catalase). They can also act directly by scavenging hydroxyl radicals, as is the case for raffinose, galactinol and fructans [17,18,19,20].

Changes in polyamine levels, including putrescine (Put), spermidine (Spd), spermine (Spm) and cadaverine (Cad), have been observed in plants subjected to different abiotic stresses such as drought, salinity, low and high temperature, nutrient deficiency and metal toxicity [21,22,23]. Polyamines are low-molecular-weight aliphatic nitrogen compounds positively charged at physiological pH. They have functional properties similar to those of osmolytes, such as hydrophobicity, protection of macromolecules, maintenance of intracellular pH and modulation of oxidative injuries [21,22,24]. For instance, treatment of barley seedlings with spermidine decreased stress-induced catalase and guaiacol peroxidase activity [25]. Spermidine also probably inhibits stress-induced activation of the microsomal NADPH oxidases and in turn ROS accumulation in cucumber cultivars [26]. In addition to their protective effects against abiotic stresses, polyamines are linked to a wide range of plant growth and developmental processes such as cell division and elongation, vascular differentiation, embryoid formation in tissue cultures, root growth, adventitious shoot formation, flower and fruit development, and leaf senescence [22,27].

To improve crop performance under adverse environmental conditions, the use of plant biostimulants has been promoted for their ability to mitigate the effects of abiotic stresses on plants [28]. Plant biostimulants are defined as any substance or microorganism applied to plants with the aim to enhance nutrition efficiency, abiotic stress tolerance and/or crop quality traits, regardless of its nutrients content [29]. Plant biostimulants include different natural substances: humic and fulvic acids, seaweed extracts, protein hydrolysates, beneficial chemical elements (i.e., silicon), inorganic salts (phosphite), chitin and chitosan derivates, beneficial fungi and plant-growth-promoting rhizobacteria [30].

Concerning protein hydrolysates, published reports conclude that they could affect the C (Carbon) and N (Nitrogen) metabolism, nutrient availability, the hormonal balance and could interact with the rhizosphere microbiome [31,32]. For instance, application of amino acid and peptide mixtures from poultry feather waste on banana fields increased the contents in phenolic compounds and flavonoids in the fruit and vegetative parts [33]. Application of a non-protein amino-acid derivative–L-pyroglutamic acid, also named pidolic acid, improved the drought tolerance of lettuce by stimulating photosynthesis, antioxidant processes and preserving the osmotic balance and the water balance [34]. Foliar applications of the legume-derived protein hydrolysate Trainer^®^ enhanced yield, the photosynthetic status, the quality attributes and the antioxidant activity of perennial wall rocket (*Diplotaxis tenuifolia*) leaves over autumn-to-spring crop cycles [35]. Moreover, following the application of this same protein hydrolysate, unfertilized lettuce showed similar yield to lettuce amended with 10 kg ha^−1^ of nitrogen, and better protection against stress, evaluated by higher lipophilic and hydrophilic antioxidant activities [36].

More research is needed to clarify the mechanisms of action of protein hydrolysates in relation to their amino acid profile and concentration, their highly variable free amino acid content due to the nature of the protein source and the processing method (e.g., low or extensive hydrolysis) [32]. In order to shed light on the mode of action of protein hydrolysates, we evaluated the effects of a commercially available free amino acid-rich hydrolysate (namely a protein hydrolysate containing a high content of free amino acids) –Leafamine^®^ (BCF Life Sciences, Pleucadeuc, France)—on water stress mitigation. We used lettuce (*Lactuca sativa* L.) as a model plant because its shallow root system makes it extremely susceptible to water deficit [37] and it has a high leaf water content [34]. Then, we compared the physiological and metabolomic responses of Leafamine^®^-treated and untreated lettuce plants to water deficit.

## 2. Results

### 2.1. Effect of Leafamine^®^ on Shoot and Root Biomass

The average shoot and root biomasses of the lettuce plants collected at the end of the three independent experiments are shown in Table 1. Shoot fresh biomass was significantly affected by the treatment, the water condition, and by the interaction of these two factors (*p* < 0.001). In untreated lettuce, water deficit decreased shoot fresh biomass by 26%, showing that water deficit was actually experienced by lettuce. Leafamine^®^ treatment significantly increased shoot fresh biomass, whether lettuce was grown under WW or WD conditions (+40% and +20%, respectively) (Table 1). Leafamine^®^ treatment and the water condition also significantly affected (*p* < 0.001) root fresh biomass, but the interaction of the two factors did not. Water deficit resulted in a decline in root fresh biomass (−29%), irrespective of the Leafamine^®^ treatment. Leafamine^®^ application also caused a decrease in root fresh biomass regardless of the water condition (−21%). These results reveal that Leafamine^®^ application significantly increased the shoot-to-root ratio (fresh biomass) of lettuce (+58%), irrespective of the water condition. In terms of root and shoot dry biomass, the treatment and water condition effects were both significant but the interaction between the two factors was not. Independent of the water condition, Leafamine^®^-treated lettuce displayed increased shoot dry biomass (+12%) and decreased root dry biomass (−25%) compared to untreated lettuce. As for the water condition effect, WD caused a significant decrease in root and shoot dry biomass (−25% and −32%, respectively), regardless of the treatment. The shoot-to-root dry biomass ratio was significantly higher in Leafamine^®^-treated lettuce, i.e., +79% and + 43% compared with the untreated WW and untreated WD plants, respectively. The “treatment x water condition” interaction was also significant (*p* < 0.05). Regarding the water content, significant differences were found for the treatment factor and the water condition factor (*p* < 0.05) taken separately (Table 1).

As a whole, Leafamine^®^ treatment increased the shoot-to-root biomass ratio even in WD conditions. Moreover, these effects were robust across three independent experiments conducted year-round. For the sake of clarity and efficacy, we focused on one representative trial (experiment 2) to decipher the effects of the biostimulant on the morphological, physiological and biochemical responses of lettuce.

### 2.2. Effect of Leafamine^®^ on Morphological Parameters

**Figure 1:**
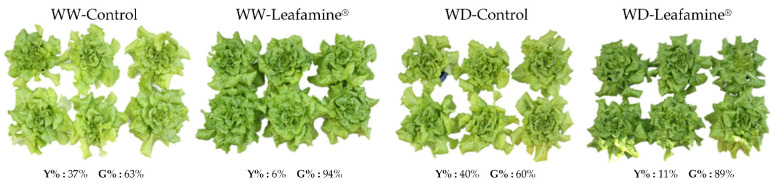
Leafamine^®^ and water condition effects on appearance of lettuces at harvest. Analysis of appearance of lettuces treated and untreated with Leafamine^®^ (0.585 g/pot) under well-watered and water-deficit conditions. **Y%** and **G%** correspond to the average percentage of “yellow color” and “green color”, respectively, of lettuces for each modality. Abbreviations: WW-Control, Untreated lettuces under well-watered conditions; WW-Leafamine^®^, Leafamine^®^-treated lettuces under well-watered conditions; WD-Control, Untreated lettuces under water-deficit conditions; WD-Leafamine^®^, Leafamine^®^-treated lettuces under water deficit conditions.

Lettuce appearance 38 days after transplanting (DAT) was clearly different between the Leafamine^®^-treated and untreated plants. Leafamine^®^-treated lettuce looked greener and larger than untreated lettuce, irrespective of the water condition (Figure 1). No interaction between the two factors on projected leaf area was denoted 21 and 34 DAT (Table 2), whereas the effect of the water condition was already significant (*p* < 0.001) 21 DAT and even higher 34 DAT. The projected leaf area was lower under WD than under WW conditions (Table 2). Leaving Leafamine^®^ treatment aside, the decrease reached −35% 34 DAT (Table 2). At that same time point, the projected leaf area of Leafamine^®^-treated lettuce was significantly higher (+16%; *p* < 0.01) than the projected leaf area of untreated lettuce (Table 2).

By contrast, Leafamine^®^ did not affect the number of produced leaves, whatever the water condition (Figure S1). The increase in shoot fresh and dry biomass of Leafamine^®^-treated lettuce (Table 1) seemed more likely associated with an increase in leaf area (Table 2) rather than greater leaf production (Figure S1), irrespective of the water condition.

### 2.3. Effect of Leafamine^®^ on Leaf Gas Exchange Parameters

**Figure 2:**
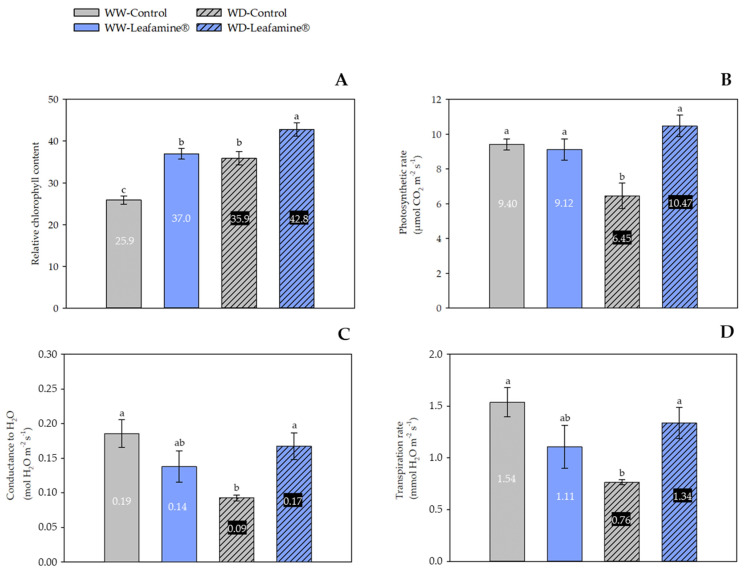
Leafamine^®^ and water condition effects on chlorophyll-relative content and leaf gas-exchange parameters; chlorophyll-relative content (**A**), net photosynthetic CO_2_ assimilation (**B**), conductance to H_2_O (**C**) and transpiration rate (**D**). Data are expressed as means ± standard errors of measures taken 36 days after transplanting, from lettuces treated and untreated with Leafamine^®^ (0.585 g/pot) grown under well-watered and water-deficit conditions (n = 4 lettuces). Letters stand for significantly different statistical classes (two-way ANOVA, *p* < 0.05 with post-hoc Tukey test). Each label value represents mean value of each measurement for each modality. Abbreviations: WW-Control, Untreated lettuces under well-watered conditions; WW-Leafamine^®^, Leafamine^®^-treated lettuces under well-watered conditions; WD-Control, Untreated lettuces under water-deficit conditions; WD-Leafamine^®^, Leafamine^®^-treated lettuces under water-deficit conditions.

The relative chlorophyll content was monitored 36 DAT. Leafamine^®^ treatment significantly enhanced the relative chlorophyll content of lettuce, irrespective of the water condition (Figure 1 and Figure 2A). The increase was 43% compared with untreated WW plants and 19% compared with untreated WD plants (Figure 2A). In addition, the percentage of “green color” is higher in Leafamine^®^-treated lettuces (Figure 1).

Furthermore, WD negatively affected the photosynthetic rate of untreated lettuce (−31%), probably due to stomatal closure, hence reduced stomatal conductance g_s_ (−53%) and a lower transpiration rate (−51%) (Figure 2B–D). Interestingly, this effect was significantly mitigated by Leafamine^®^ application: the photosynthetic rate, stomatal conductance and the transpiration rate of WD Leafamine^®^-treated were not significantly different than those of WW lettuce, whether treated or untreated. Under WD conditions, the photosynthetic rate, stomatal conductance and the transpiration rate were higher in Leafamine^®^-treated lettuce than in untreated lettuce (+62%, +89% and +76%, respectively) (Figure 2B–D).

### 2.4. Effect of Leafamine^®^ on the Leaf Carbon, Nitrogen and Sulfur Contents

**Figure 3:**
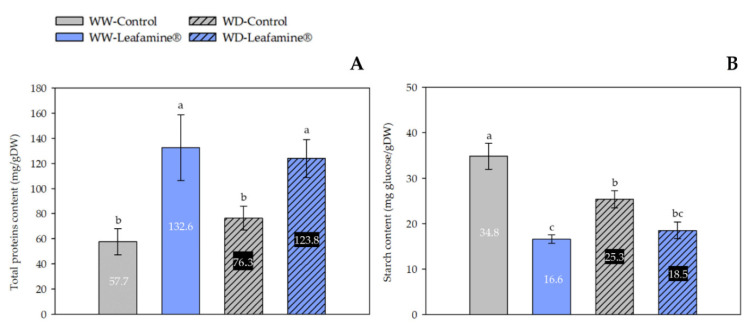
Leafamine^®^ and water condition effects on leaf total protein (**A**) and starch contents (**B**). Data are expressed as means ± standard errors of measures taken 30 days after transplanting, from lettuces treated and untreated with Leafamine^®^ (0.585 g/pot) grown under well-watered and water- deficit conditions (n = 5 lettuces). Letters stand for significantly different statistical classes (two-way ANOVA, *p* < 0.05 with post-hoc Tukey test). Each label value represents mean value of each measurement for each modality. Abbreviations: WW-Control, Untreated lettuces under well-watered conditions; WW-Leafamine^®^, Leafamine^®^-treated lettuces under well-watered conditions; WD-Control, Untreated lettuces under water-deficit conditions; WD-Leafamine^®^, Leafamine^®^-treated lettuces under water-deficit conditions.

The leaf total protein and starch contents were monitored 30 DAT (Figure 3A,B), after four applications of Leafamine^®^ or water (control). The leaf elemental contents in carbon, nitrogen and sulfur and intrinsic water use efficiency were determined one week later, at harvest (Table 3).

Under WD and WW conditions, Leafamine^®^ induced a significant increase in leaf total protein content (+62% and 2.3 fold, respectively) compared to untreated lettuce (Figure 3A). WD caused a significant decrease in the leaf starch content of untreated lettuce (−27%) compared with WW untreated lettuce. Moreover, Leafamine^®^ application significantly decreased the leaf starch content: WW × Leafamine^®^ and WD × Leafamine^®^ lettuce plants exhibited the lowest leaf starch contents. The decrease was 52% compared with untreated WW plants and 27% compared with untreated WD plants (Figure 3B).

**Figure 4:**
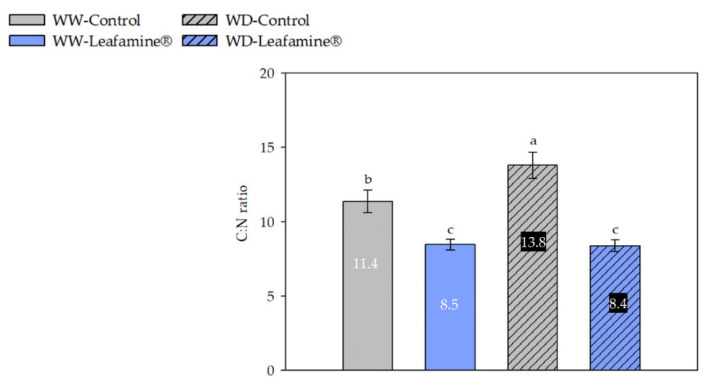
Leafamine^®^ and water condition effects on leaf C:N ratio. Data are expressed as means ± standard errors of measures taken 38 days after transplanting, from lettuces treated and untreated with Leafamine^®^ (0.585 g/pot) grown under well-watered and water-deficit conditions (n = 5 lettuces). Letters stand for significantly different statistical classes (two-way ANOVA, *p* < 0.05 with post-hoc Tukey test). Each label value represents mean value of each measurement for each modality. Abbreviations: WW-Control, Untreated lettuces under well-watered conditions; WW-Leafamine^®^, Leafamine^®^-treated lettuces under well-watered conditions; WD-Control, Untreated lettuces under water-deficit conditions; WD-Leafamine^®^, Leafamine^®^-treated lettuces under water-deficit conditions.

The elemental carbon content, representing structural (e.g., cellulose) and non-structural (e.g., soluble sugars, starch) carbohydrates did not differ between “treatment” and “water condition” (Table 3). In accordance with the increase in leaf protein content in Leafamine^®^-treated lettuce observed 30 DAT (Figure 3A), the elemental nitrogen content was only significantly different for “treatment”, and was higher in Leafamine^®^-treated (+45%) than in untreated lettuce. Consistently, the leaf C:N ratio decreased in the leaves of Leafamine^®^-treated lettuce under both water conditions, with a significant interaction between “treatment” and “water condition”: −25% and −39% between Leafamine^®^-treated and untreated lettuce under WW and WD conditions, respectively (Figure 4). Regarding the elemental sulfur content, “treatment” and “water condition” taken separately were significant (*p* < 0.05). Leafamine^®^ application resulted in a significant increase (+31%) of the leaf sulfur content, whereas WD decreased it (−19%).

Finally, only “water condition” had a significant impact (*p* < 0.01) on intrinsic water use efficiency, which increased in lettuce under WD (+22%), irrespective of the treatment (Table 3).

### 2.5. Leafamine^®^ on Leaf Defense and Growth Phytohormones

The endogenous hormone content was measured in leaves of lettuce plants, treated or untreated with Leafamine^®^ four times during the cultivation period. Four hormones were analyzed: abscisic acid, salicylic acid, jasmonic acid and zeatin-o-glucoside ribose (Table 4). As expected, WD significantly increased the abscisic acid content (34.2 fold; *p* < 0.001) of the leaves of WD lettuce compared with WW lettuce. Leafamine^®^ also globally increased the ABA content (2.8 fold), mainly explained by the values found under WD. Morever, the “treatment × water condition” interaction was highly significant: Leafamine^®^ application increased the ABA content 1.7 fold in WD lettuce, and 11.2 fold in WW lettuce (Table 4). The leaf endogenous salicylic acid content was only significantly affected by the “treatment” factor (*p* < 0.05) that was higher (+86%) in Leafamine^®^-treated lettuce, independently of the water condition Finally, the zeatin-o-glucoside ribose content was significantly impacted by “treatment”, “water condition” and the interaction between the two factors. It increased in the leaves of Leafamine^®^-treated WW lettuce (+33% compared with untreated WW lettuce) and WD conditions (+61% compared with untreated WD lettuce) (Table 4).

### 2.6. Effect of Leafamine^®^ on Metabolomic Profiling

**Figure 5:**
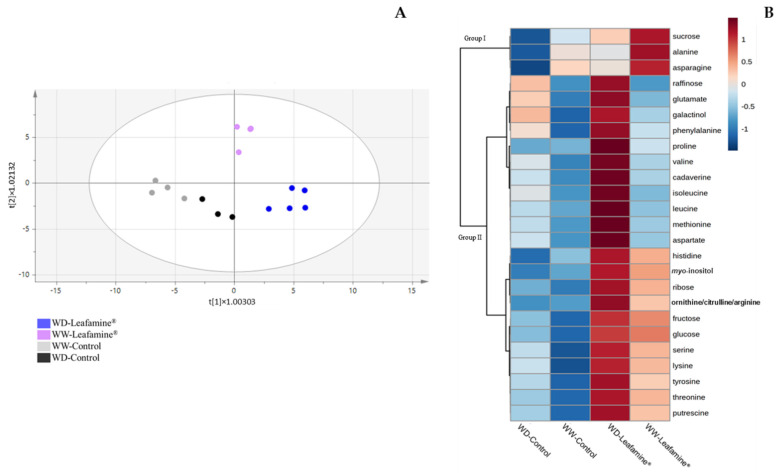
Leafamine^®^ and water condition effects on metabolomics pattern of lettuce leaves at harvest. (**A**) Score plot of the multivariate analysis by Orthogonal Projection to Latent Structures (OPLS) using water condition and treatment as predicted Y variables, and metabolites detected 38 DAT as predicting X × variables. (**B**) Heat map showing average level of the most discriminating metabolites (25) at harvest (38 DAT) for each modality, with a hierarchical clustering on left (Pearson correlation). Abbreviations: WD-Leafamine^®^, Leafamine^®^-treated lettuces under water-deficit conditions; WW-Leafamine^®^, Leafamine^®^-treated lettuces under well-watered conditions; WW-Control, ntreated lettuces under well-watered conditions; WD-Control, Untreated lettuces under water-deficit conditions.

**Figure 6:**
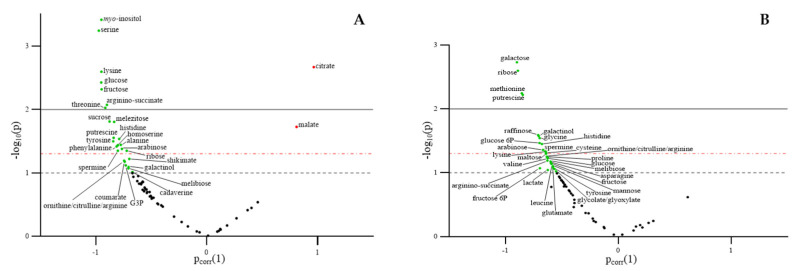
Metabolites identified in leaves of well-watered and deficit-irrigated lettuces at harvest. Volcano plot illustrating identified metabolites (Leafamine^®^ application vs. control) in leaves of lettuces grown under well-watered (**A**) and water-deficit conditions (**B**) at harvest (38 DAT), with the *p*-value (*y*-axis) and the loading in the OPLS-DA (using treatment as ClassID, for each water condition) (*x*-axis). Coloured dots represent differentially expressed metabolites with statistical significance at *p*-value < 0.1 and 0.05 (overhead horizontal black and red dotted line, respectively) and *p*-value < 0.01 (overhead horizontal black continuous line). Green and red dots represent metabolites that are up-expressed and down-expressed in Leafamine^®^-treated and untreated lettuces, respectively. Abbreviations: G3P, glyceraldehyde 3-phosphate; glucose 6P, glucose 6-phosphate; fructose 6P, fructose 6-phosphate.

Metabolomic profiling was carried out on leaves collected 20 DAT (Figure S2) and at harvest (Figure 5 and Figure 6). To better understand the effects of Leafamine^®^ on WD and WW lettuce plants at harvest, a supervised multivariate analysis was performed, considering the two factors (treatment and water condition). The OPLS (Figure 5A) showed a predictive (R^2^Y = 0.87), robust (Q^2^ = 0.741) and significant (P_CV-ANOVA_ = 0.03 and 0.02, for water condition and treatment, respectively) model, that separated “WW-untreated” from “WW-Leafamine^®^”, as well as “WD-Control” from “WD-Leafamine^®^”. As evidenced by the heat map representing the most discriminating metabolites (Figure 5B), two major groups of compounds were induced by Leafamine^®^.

Group I (e.g., sucrose) was induced by Leafamine^®^ and repressed by WD, whereas group II (e.g., galactinol and putrescine) was induced to a greater or lesser degree by both Leafamine^®^ and WD.

For each water condition, the metabolites detected in lettuce leaves at harvest (38 DAT) were combined into a volcano plot to identify differentially accumulated metabolites between Leafamine^®^-treated and untreated lettuce plants (Figure 6A,B). At harvest, higher levels of amino acids–serine, lysine, threonine (*p* < 0.01), histidine, tyrosine, homoserine, alanine, phenylalanine (*p* < 0.05) and ornithine/citrulline/arginine (*p* < 0.1) –were found in WW Leafamine^®^-treated lettuce. In these plants, the levels of polyamines, putrescine, spermine (*p* < 0.05) and cadaverine (*p* < 0.1) also rose. Moreover, the levels of soluble sugars—glucose, fructose (*p* < 0.01) and sucrose (*p* < 0.05)—were also significantly enhanced in WW Leafamine^®^-treated lettuce (Figure 6A). This result was in accordance with the decreased leaf starch content found in WW Leafamine^®^-treated lettuce (Figure 3B).

Under WD, significantly higher levels of galactose, ribose, methionine and putrescine were found in Leafamine^®^-treated lettuce (*p* < 0.01). Several amino acids—glycine, histidine, lysine, cysteine (*p* < 0.05), ornithine/citrulline/arginine, proline, valine, asparagine, leucine, tyrosine (*p* < 0.1) and the polyamine spermine (*p* < 0.05)—were more abundant in WD Leafamine^®^-treated lettuce. Glucose 6P (*p* < 0.05), glucose, fructose 6P and fructose (*p* < 0.1) were also detected in greater amounts in these plants. The raffinose and galactinol contents significantly increased (*p* < 0.05) in WD Leafamine^®^-treated lettuce (Figure 6B).

## 3. Discussion

Persistent abiotic stress conditions such as drought are major adverse environmental factors that negatively affect the physiology and biochemistry of plants and limit crop production worldwide [38]. In order to mitigate these negative effects, many research works have been focused on the use of plant biostimulants because they represent a commonly used environment-friendly strategy [39]. Lettuce is widely considered to be an ideal plant model for evaluating the protective effect of biostimulants on vegetative growth [14,40]. In line with this, we characterized the effects of Leafamine^®^, a free amino acid-rich biostimulant, on the physiological performance of lettuce plants grown under two contrasting water regimes (well-watered and water deficit) for 38 days. Leafamine^®^ supply to lettuce roots was associated with a better growth performance and resilience to water stress compared to the untreated plants. This protective effect is associated with morphological, biochemical and hormonal modifications.

Leafamine^®^ supplementation to the root system rose the level of putrescine as early as 20 DAT (Figure S2), and this effect persisted until the end of the cultivation period irrespective of the water regime. The leaves of Leafamine^®^-treated plants also exhibited an elevated level of many intermediate metabolites of the putrescine synthesis pathway such as glutamate and arginino-succinate, whereas those associated with putrescine degradation (i.e., GABA and succinate) were not significantly affected compared to untreated plants [41,42]. Putrescine belongs to the polyamines, which induce tolerance against abiotic stresses, including during water deficit [21,22]. For instance, putrescine application on *Thymus vulgaris* L. mitigates the impacts of drought, by causing accumulation of total soluble phenolic compounds and activities of some related enzymes [43]. In addition, Leafamine^®^ supplementation was accompanied by a high shoot biomass at the expense of root biomass resulting in an elevated shoot-to-root ratio compared to the control. Yet, these morphological shoot and root changes are a hallmark of the putrescine effect, that its accumulation resulted in primary root length in both Arabidopsis [44,45] and *Pringlea antiscorbutica* [46]. Leafamine^®^ also induced starch degradation along with significant accumulation of hexoses in lettuce leaves. This starch remobilization is considered as an essential process for plant performance under challenging environmental conditions, through the release of energy, sugars and derived metabolites that help crops mitigate the stress conditions [47]. A reduction of number of leaf starch granules was reported in cucumber seedlings in response to putrescine application through the downregulation of starch synthesis enzymes (AGPase, ADPgluco-pyrophosphorylase), and the upregulation of a starch-degrading enzyme (β-amylase) [48]. As Leafamine^®^ does not contain putrescine, its synthesis could be endogenously triggered by this kind of biostimulant in lettuce. In consistence with this, it is unlikely that arginine—a component of Leafamine^®^—may explain the increased putrescine level detected in Leafamine^®^-treated lettuce, because leaf arginine spraying only had minor effects on the polyamine levels of sugarcane plants subjected to water deficit [49]. It has been observed that vegetal-derived protein hydrolysates treatment caused accumulation of polyamines (namely sinapoyltyramine and triferuloyl spermidine) in tomato plants [50]. Moreover, similar trends have been reported for melatonin, a low-molecular-weight indoleamine involved in various biological processes and responses to environmental cues in plants. Exogenous application of melatonin increased the polyamine contents by accelerating the metabolic flow from the precursor amino acids arginine and methionine to polyamines and decreasing their degradation under physiological and stress conditions [51]. Since putrescine is also considered as a nitrogen and carbon store for plants, we speculate that Leafamine^®^ reprograms the intracellular metabolism by promoting the flow of carbon into the nitrogen metabolism, thereby altering the carbon/nitrogen ratio. This assumption is corroborated by the increased levels of organic acids (malate, citrate) measured in untreated plants, and of amino acids (glycine, valine, methionine, arginino-succinate and glutamate) in Leafamine^®^-treated plants. Although these findings suggest a likely role of putrescine in the action of Leafamine^®^ in plants, further research are required to establish the molecular link between Leafamine^®^ and putrescine accumulation in Leafamine^®^-treated lettuces.

The action of Leafamine^®^ supplementation was not limited to putrescine as it triggered ABA accumulation in lettuce leaves at varying degrees, irrespective of the water regime. Confirming this result, application of amino acids based biostimulant increased ABA content of grapevine under drought-stressed conditions [52]. ABA is well known to be involved in plant responses to abiotic stresses [53,54] and plays a key role in regulating genes expression, proteins and enzymatic activities behind plant cell dehydration tolerance [55,56]. Furthermore, a positive feedback between ABA and putrescine was interestingly evidenced when ABA accumulated through upregulation of 9-cis-epoxycarotenoid dioxygenase (NCED) in plants overexpressing arginine decarboxylase and genes of putrescine synthesis [57]. Such a connection between Leafamine^®^, putrescine and ABA was boosted under WD: Leafamine^®^-treated lettuce plants showed a high level of ABA and putrescine compared to WD untreated lettuce plants, together with increased photosynthetic activity, chlorophyll content and biomass, and increased levels of other protective molecules, mostly osmolytes (proline and soluble sugars), which are necessary to maintain lower water potential and to limit damages caused by water shortage [13]. Overall, putrescine application under abiotic stress improves the photosynthetic capacity by increasing the photochemical efficiency of PSII in cucumber seedlings [58], the water status and the chlorophyll, proline, amino acid and soluble sugar contents in wheat [59]. Moreover, ABA accumulation is necessary to induce proline accumulation in Arabidopsis seedlings at low water potential [60] and ABA treatment protects photosynthetic apparatus of two Arabidospis genotype (wild-type and ABA-deficient mutant) leaves from drought-induced damage during 24 h [61]. We cannot rule out that putrescine and ABA act synergistically to confer a better tolerance of lettuce to water stress. Additional investigations are required in the future to investigate this hypothesis.

Leafamine^®^ can also improve lettuce tolerance to WD by eliciting the accumulation of raffinose and its direct precursor galactinol, compared to untreated lettuce. Raffinose and galactinol have been massively studied as they protect plant cells in response to abiotic stresses, including when plants encounter drought stress [18,20,62]. Galactinol is synthesized by UDP-D-Gal:*myo*-inositol (1-α-_D_) galactosyltransferase (GS; EC 2.4.1.123), which catalyzes the reaction; UDP-Gal + *myo*-inositol → galactinol + UDP [63]. Besides galactinol and raffinose, WD Leafamine^®^-treated lettuce plants displayed an elevated level of galactose. Galactose-1-phosphate is the first precursor for the synthesis of UDP-Galactose catalyzed by galactose-1-*p* uridylyltransferase [64]. By contrast to putrescine, Leafamine^®^-mediated raffinose accumulation was limited to the WD plants (Figure 6B), indicating that Leafamine^®^ application may trigger different mechanisms involved in the accumulation of protective molecules. One future task will be to understand the molecular mechanism behind Leafamine^®^-mediated raffinose accumulation.

## 4. Materials and Methods

### 4.1. Plant Material, Growth Conditions and Harvesting Procedure

Three independent experiments were carried out in a greenhouse located in Angers (Research Institute of Horticulture and Seeds, France) between April 2019 and November 2020. During that period, the average greenhouse temperature was 23.4 ± 6.1 °C during the day and 15.3 ± 4.6 °C at night. The average relative humidity ranged between 51.0% and 67.0%. The average day length was 14 h 20 min. Eighteen-day old lettuce (*Lactuca sativa* L. var. *Icaro*) seedlings were transplanted in 1.5 L plastic pots containing professional (peltrAcom 113AT) potting soil (black and blond peat, pH = 6–6.5; EC (µS/cm) = 270–500). The potting soil was supplemented with a starter fertilizer (14:16:18 N:*p*:K and trace elements; 1 kg/m^3^). Foliar fertilizer Agroleaf power^®^ was added to the lettuce plants three times for each trial to avoid nutrient stress. The lettuce plants were arranged in a randomized design for each experiment. A combination of two factors was tested: water condition (plants under well-watered and water deficit conditions: WW and WD, respectively) and treatment (untreated plants and plants treated with the biostimulant Leafamine^®^: control and Leafamine^®^, respectively). The numbers of lettuce plants included in each experiment are reported in Table S1. Concerning the water condition factor, the water deficit was monitored by weighing the pots. For the lettuce plants grown under WW conditions, water was supplied to maintain the substrate close to 100% water-holding capacity (WHC). For the lettuce plants grown under WD conditions, water was supplied so as to maintain WHC at 70%.

Regarding the treatment factor, Leafamine^®^ produced from extensive chemical hydrolysis of poultry feathers was provided by BCF Life Sciences. One of the specificities of Leafamine^®^ is to provide a unique and stable amino acid profile exclusively under L form, with 81.5% of free amino acid content (Tables S2 and S3, Figure S3). This mix of free amino acids is composed of serine (11.3%), proline (9.8%), glutamic acid (9.6%), glycine (7.3%), aspartic acid (6.7%), leucine (6.1%), arginine (5.3%), valine (5.0%), alanine (4.5%), threonine (4.4%), phenylalanine (4.2%), isoleucine (3.1%), cystine (1.1%), lysine (1.5%), tyrosine (0.6%), histidine (0.6%) and methionine (0.4%). Leafamine^®^ is also composed of 6% of small peptides (Table S4). This biostimulant is highly soluble and has a very low molecular weight (100% less than 800 Dalton) to make uptake by plants easier. The biostimulant (0.585 g plant^−1^) was applied to lettuce roots. One hundred and five mL per plant of a solution containing 5.57 g.L^−1^ of Leafamine^®^ was applied to the substrate, whereas 105 mL of water was applied to the control plants. The Leafamine^®^ dose was established in compliance with the manufacturer’s recommendations. The treatments started at the third true leaf stage and were repeated weekly three times. Twenty and 30 days after transplanting (after 3 and 4 treatments, respectively), and at harvest, young fully developed fourth leaves were sampled at midday (one leaf per plant), immediately frozen in liquid nitrogen and kept at −80 °C to be used for metabolomic analyses (Figure S4). At the end of each experiment (38 days after transplanting), shoot fresh biomass was measured by weighing the whole aerial part of the lettuce plants. Root fresh biomass was also assessed after substrate withdrawal. Shoots and roots were oven-dried at 70 °C to a constant weight to determine shoot and root dry biomasses.

The plant water content (WC) was calculated as follows: WC = [(FW − DW)/FW] × 100; where FW = fresh weight, DW = dry weight [65]. To assess the average projected leaf area, the lettuce plants were photographed in top view 21 and 34 days after transplanting. The projected leaf area was calculated by analyzing the photographs using the threshold color plugin from ImageJ software (http://imagej.nih.gov/ij/ accessed on 29 September 2021).

### 4.2. Elemental C, N and S Content Measurements

At the end of the experiment, leaf samples were ground in a mortar in liquid N_2_, lyophilized and 3 mg was used to determine the total nitrogen, carbon and sulfur contents as well as the relative isotopic abundance of ^15^N, ^13^C and ^34^S using elemental analyzer combustion isotope ratio mass spectrometry (vario PYRO cube^®^ coupled to isoprime precisION). Intrinsic water use efficiency was calculated, by neglecting the mesophyll conductance (g_m_), as follows [66]:

$$\frac{A}{\mathrm{gs}}=1.6\times \mathrm{Ca}\frac{b-\Delta }{\left(b-a\right)}$$

where A and g_s_ are the assimilation rate and stomatal conductance, respectively; c_a_, a, b and Δ are the atmospheric CO_2_ concentration; and ^13^C discrimination during CO_2_ diffusion through stomata, fractionation due to carboxylation and net fractionation during photosynthesis that can be calculated from the isotope composition (δ^13^C) of atmospheric CO_2_ and plant fixed carbon [67,68], respectively. The iWUE_sim_ model [66] was used for this measure.

### 4.3. Gas Exchange and Chlorophyll Content Measurements

Gas exchanges were measured on four plants per condition using a gas exchange open system Li-Cor 6400 xt with a 6 cm^2^ fluorescence chamber (Li-Cor, Austin, TX, USA).

Net photosynthesis (A), stomatal conductance (g_s_) and the transpiration rate (E) were recorded simultaneously and measured under typical conditions (400 µmol mol^−1^ CO_2_, leaf temperature 20 °C, 300 µmol.m^−2^s^−1^ PAR and 55% relative humidity). The measurements were performed between 9 a.m. and 1 p.m., 36 days after transplanting.

The chlorophyll content was estimated 36 days after transplanting with the soil plant analysis development (SPAD) index determined on the middle part of a young fully expanded leaf, using a SPAD-502 chlorophyll meter.

### 4.4. Starch Content Measurement

The analysis was carried out as described previously [69].

### 4.5. Total Protein Content Measurement

Five mg of lyophilized leaf powder were homogenized with 1.3 mL of 80% aqueous ethanol at 80 °C for 30 min, and then with 700 µL of 50% aqueous ethanol at 80 °C for 30 min. The resulting suspension was centrifuged at 12,000× *g* and 4 °C for 10 min. The pellet was used for protein analysis. It was resuspended in 400 µL of NaOH 0.1 M, and the suspension was heated at 95 °C for 30 min and cooled at room temperature. Then, it was centrifuged at 2500 rpm and 4 °C for 5 min. Total proteins were determined in the supernatant spectrophotometrically using SPECTROstar Nano^®^ and bovine serum albumin as a standard.

### 4.6. Phytohormone Content Measurements

For each sample, 2 mg of dry powder was extracted with 0.8 mL of acetone/water/acetic acid (80/19/1 v:v:v). Stable labeled isotopes of abscisic acid, salicylic acid, jasmonic acid, indole-3-acetic acid and cytokinins were used as internal standards and prepared as described previously [70]. One ng of each standard (0.5 ng for cytokinins) was added to the sample. The extract was vigorously shaken for 1 min, sonicated at 25 Hz for 1 min, shaken in a Thermomixer at 10 °C for 10 min (Eppendorf^®^) and then centrifuged (8000× *g*, 10 °C, 10 min). The supernatants were collected, and the pellets were re-extracted twice with 0.4 mL of the same extraction solution, vigorously shaken (1 min) and sonicated (25 Hz; 1 min). After the centrifugations, the three supernatants were pooled and dried (final volume 1.6 mL). Each dry extract was dissolved in 100 µL of acetonitrile/water (50/50 v:v), filtered and analyzed using a Waters Acquity ultra performance liquid chromatograph coupled to a Waters Xevo Triple quadrupole mass spectrometer TQS (UPLC-ESI-MS/MS). The compounds were separated on a reverse-phase column (Uptisphere C18 UP3HDO, 100 × 2.1 mm × 3 µm particle size; Interchim, France) at a flow rate of 0.4 mL min^−1^ and using a binary gradient: (A) acetic acid 0.1% in water (v:v) and (B) acetonitrile with 0.1% acetic acid. The column temperature was 40 °C. The following binary gradient was used for abscisic acid, salicylic acid, jasmonic acid and indole-3-acetic acid (time, % A): (0 min, 98%), (3 min, 70%), (7.5 min, 50%), (8.5 min, 5%), (9.6 min, 0%), (13.2 min, 98%), (15.7 min, 98%). The following gradient was used for cytokinins (time, % A): (0 min, 95%), (13 min, 40%), (16 min, 0%), (16.5 min, 95%). Mass spectrometry was conducted in electrospray and multiple reaction monitoring scanning (MRM) mode, in positive ion mode for indole-3-acetic acid and in negative ion mode for the other hormones. The relevant instrumental parameters were set as follows: capillary 1.5 kV (negative mode), source block and desolvation gas temperatures 130 °C and 500 °C, respectively. Nitrogen was used to assist the cone and desolvation (150 L h^−1^ and 800 L h^−1^, respectively), and argon was used as the collision gas at a flow rate of 0.18 mL min^−1^.

### 4.7. Metabolomics Analysis

Gas chromatography coupled to mass spectrometry was carried out with a 436-GC coupled to a Simple Quadruple (SQ) SCION MS (Bruker^®^). The column was an RTX-5 w/integra-Guard (30 m × 0.25 mm i.d. + 10-m integrated guard column; Restek). Leaf samples were ground in a mortar in liquid N_2_ (30 mg of fresh powder) and then in 1 mL of 80% methanol in which ribitol (100 μmol L^−1^) was added as an internal standard. The extracts were transferred to 2-mL Eppendorf tubes and centrifuged at 10,000× *g* and 4 °C for 15 min. The supernatants were SpeedVac dried and stored at −80 °C until analysis. Methoxyamine was dissolved in pyridine at 50 mg mL^−1^, and 40 μL of this mixture was used to dissolve the dry individual samples. Following vigorous mixing, the samples were incubated at 37 °C under shaking for 90 min. Forty μL of N-methyl-N-(trimethyl-silyl) trifluoroacetamide was added, and the mixture was vortexed and incubated at 37 °C under shaking for 30 min. Before loading into the gas chromatography autosampler, a mix of a series of fourteen alkanes (chain lengths C10–C36) was included. Analyses were performed by injecting 1 μL in splitless or split mode at 280 °C injector temperature. Separation was performed in helium as a carrier gas at 1 mL min^−1^ in constant flow mode and using a temperature ramp from 70 °C to 320 °C between 4 and 22 min, followed by 5 min at 320 °C. Ionization was achieved by electron impact at 70 eV, and the mass spectrum acquisition rate was 20 spectra s^−1^ over the 70–500 m/z range. Peak identity was established by comparing the fragmentation pattern with the mass spectra from our homemade metabolite spectrum database and from another available database (National Institute of Standards and Technology), and based on the retention index using the alkane series as retention standards. The peaks were integrated using Bruker^®^ MS Workstation software.

### 4.8. Statistical Analyses

All statistical tests were performed using R statistical software (R Foundation for Statistical Computing, Vienna, Austria). Apart from metabolomic profiling data, data were compared using two-way ANOVA (Treatment and water condition, as factors), followed by Tukey post-hoc test.

Metabolomics data were loaded into SIMCA 17 (Unmetrics, Malmö, Sweden) and Orthogonal Projections to Latent Structures (OPLS) was carried out, using water condition and treatment as predicted Y variables, and metabolites as predicting X variables. The performance of the multivariate model was assessed using the determination of goodness-of-fit R^2^ and goodness-of-prediction Q^2^Y. CV-ANOVA was applied to validate the model.

For each water condition, at harvest, metabolites were visualized with volcano plots where the logarithm of the *p*-value obtained in the t-test was plotted against the rescaled loading (p_corr_) obtained by Orthogonal Projections to Latent Structures Discriminant Analysis OPLS-DA (using treatment as ClassID, for each water condition). In this representation, the metabolites more detected in Leafamine^®^-treated lettuce had maximal −log_10_(*p*) and minimal p_corr_ values. A heat map summarizing the most representative metabolites in lettuces at harvest, with hierarchical clustering on the left (Pearson’s correlation), was drawn using the https://www.metaboanalyst.ca/online program (9 September 2021).

## 5. Conclusions

Leafamine^®^, a free amino acid-rich biostimulant, promotes shoot growth at the expense of root development, and mitigates the adverse effects of water deficit. Root application of Leafamine^®^ may act through osmotic adjustment, leading to the accumulation of amino acids (proline), carbohydrates (soluble sugars) and polyols in lettuce cells. This Leafamine^®^-dependent protective effect is complex and involves a deep metabolic adjustment in leaf–mostly related to ABA, putrescine and raffinose accumulation. One main future task will be to investigate the coordination and interaction of these regulatory pathways in the Leafamine^®^ action on plant under variable water supply conditions.

## Figures and Tables

**Table 1:** Leafamine^®^ and water condition effects on lettuce shoot and root biomasses at the harvest.

Factor	Shoot Fresh Biomass (g Plant^−1^)	Root Fresh Biomass (g Plant^−1^)	Shoot: Root *(Fresh biomass)*	Shoot Dry Biomass (g Plant^−1^)	Root Dry Biomass (g Plant^−1^)	Shoot: Root *(Dry biomass)*	Water Content (%)
**Treatment**							
Control	57.7 ± 1.4	12.1 ± 0.6	6.4 ± 0.4	5.0 ± 0.2	1.2 ± 0.1	4.0 ± 0.2	92.3 ± 0.2
	[100]	[100]	[100]	[100]	[100]	[100]	[100]
Leafamine®	75.5 ± 2.4	9.6 ± 0.5	10.1 ± 0.4	5.6 ± 0.3	0.9 ± 0.1	6.4 ± 0.3	93.3 ± 0.2
	[131]	[79]	[158]	[112]	[75]	[160]	[101]
Significance	***	***	***	*	***	***	*
n	n = 88	n = 48	n = 48	n = 48	n = 48	n = 48	n = 48
**Water condition**							
Well-watered (WW)	79.3 ± 2.1	12.6 ± 0.6	8.1 ± 0.4	6.3 ± 0.2	1.2 ± 0.1	5.4 ± 0.3	93.1 ± 0.2
	[100]	[100]	[100]	[100]	[100]	[100]	[100]
Water deficit (WD)	53.9 ± 1.1	9.0 ± 0.4	8.4 ± 0.6	4.3 ± 0.2	0.9 ± 0.0	5.1 ± 0.2	92.5 ± 0.2
	[68]	[71]	[104]	[68]	[75]	[94]	[99]
Significance	***	***	NS	***	***	NS	*
n	n = 88	n = 48	n = 48	n = 48	n = 48	n = 48	n = 48
**Treatment × Water condition**							
Control × WW	66.2 ± 1.6 b	14.0 ± 1.0	6.3 ± 0.5	5.8 ± 0.2	1.5 ± 0.1	3.8 ± 0.2 b	92.5 ± 0.3
	[100]	[100]	[100]	[100]	[100]	[100]	[100]
Leafamine® × WW	92.4 ± 2.7 a	11.3 ± 0.6	10.0 ± 0.5	6.8 ± 0.3	1.0 ± 0.1	6.8 ± 0.4 a	93.8 ± 0.2
	[140]	[81]	[159]	[117]	[67]	[179]	[101]
Control × WD	49.0 ± 1.2 d	10.1 ± 0.4	6.6 ± 0.7	4.2 ± 0.2	1.0 ± 0.0	4.2 ± 0.2 b	92.2 ± 0.2
	[74]	[72]	[105]	[72]	[67]	[111]	[100]
Leafamine® × WD	58.6 ± 1.5 c	7.8 ± 0.5	10.2 ± 0.7	4.5 ± 0.2	0.8 ± 0.1	6.0 ± 0.3 a	92.9 ± 0.2
	[89]	[56]	[162]	[78]	[53]	[158]	[100]
Significance	***	NS	NS	NS	NS	*	NS
n	n = 44	n = 24	n = 24	n = 24	n = 24	n = 24	n = 24

Data are expressed as means ± standard errors of measures taken at harvest of each experiment (38 days after transplanting), from lettuces treated and untreated with Leafamine^®^ (0.585 g/pot) grown under well-watered and water-deficit conditions. Values were compared by using two-way ANOVA (NS, *, *** non-significant or significant at 0.05 and 0.001, respectively). If Treatment × Water condition interaction was significant, data were subjected to Tukey’s multiple comparison test. Different letters indicate statistical differences for *p* < 0.05. [Base 100 index] is shown for each measurement for each modality.

**Table 2:** Leafamine^®^ and water condition effects on projected leaf area 21 and 34 days after transplanting.

Factor	Projected Leaf Area (cm^2^) *21 DAT*	Projected Leaf Area (cm^2^) *34 DAT*
**Treatment**		
Control	755.4 ± 46.7	787.3 ± 51.3
	[100]	[100]
Leafamine®	790.5 ± 41.3	910.3 ± 70.5
	[105]	[116]
Significance	NS	**
n	n = 14	n = 14
**Water condition**		
Well-watered (WW)	897.0 ± 26.2	1021.6 ± 48.7
	[100]	[100]
Water deficit (WD)	640.8 ± 18.8	667.5 ± 22.6
	[71]	[65]
Significance	***	***
n	n = 14	n = 14
**Treatment × Water condition**		
Control × WW	883.3 ± 33.4	930.5 ± 38.4
	[100]	[100]
Leafamine® × WW	910.7 ± 45.1	1112.7 ± 80.3
	[103]	[120]
Control × WD	606.3 ± 32.0	620.2 ± 23.7
	[69]	[67]
Leafamine® × WD	670.3 ± 18.3	708.0 ± 30.9
	[76]	[76]
Significance	NS	NS
n	n = 7	n = 7

Data are expressed as means ± standard errors of measures taken 21 and 34 days after transplanting (DAT) from lettuces treated and untreated with Leafamine^®^ (0.585g/pot) grown under well-watered and water-deficit conditions. Values were compared by using two-way ANOVA (NS, **, *** non- significant or significant at 0.01 and 0.001, respectively). If Treatment × Water condition interaction was significant, data were subjected to Tukey’s multiple comparison test. Different letters indicate statistical differences for *p* < 0.05. [Base 100 index] is shown for each measurement for each modality.

**Table 3:** Leafamine^®^ and water condition effects on carbon, nitrogen and sulfur elemental contents in leaves and on intrinsic water use efficiency.

Factor	Carbon Content (% Dry Weight)	Nitrogen Content (% Dry Weight)	Sulphur Content (% Dry Weight)	Intrinsic Water-Use Efficiency (µmol mol^−1^)
**Treatment**				
Control	40.18 ± 0.16	3.27 ± 0.19	0.29 ± 0.02	145.77 ± 6.96
	[100]	[100]	[100]	[100]
Leafamine^®^	39.71 ± 0.24	4.75 ± 0.13	0.38 ± 0.03	153.85 ± 9.38
	[99]	[145]	[131]	[106]
Significance	NS	***	*	NS
n	n = 10	n = 10	n = 10	n = 10
**Water condition**				
Well-watered (WW)	40.10 ± 0.21	4.17 ± 0.25	0.37 ± 0.03	134.67 ± 5.36
	[100]	[100]	[100]	[100]
Water deficit (WD)	39.80 ± 0.22	3.85 ± 0.34	0.30 ± 0.02	164.95 ± 7.43
	[99]	[92]	[81]	[122]
Significance	NS	NS	*	**
n	n = 10	n = 10	n = 10	n = 10
**Treatment × Water condition**				
Control × WW	40.45 ± 0.16	3.62 ± 0.25	0.31 ± 0.05	135.72 ± 9.27
	[100]	[100]	[100]	[100]
Leafamine^®^ × WW	39.74 ± 0.35	4.73 ± 0.21	0.43 ± 0.05	133.62 ± 7.65
	[98]	[131]	[139]	[98]
Control × WD	39.92 ± 0.25	2.93 ± 0.19	0.27 ± 0.02	155.82 ± 9.80
	[99]	[81]	[87]	[115]
Leafamine^®^ × WD	39.69 ± 0.41	4.77 ± 0.20	0.33 ± 0.02	174.08 ± 11.46
	[98]	[132]	[106]	[128]
Significance	NS	NS	NS	NS
n	n = 5	n = 5	n = 5	n = 5

Data are expressed as means ± standard errors of measures taken at harvest (38 days after transplanting) from leaves of lettuces treated and untreated with Leafamine^®^ (0.585 g/pot) grown under well-watered and water-deficit conditions. Values were compared by using two-way ANOVA (NS, *, **, *** non-significant or significant at 0.05, 0.01 and 0.001, respectively). If Treatment × Water condition interaction was significant, data were subjected to Tukey’s multiple comparison test. Different letters indicate statistical differences for *p* < 0.05. [Base 100 index] is shown for each measurement for each modality.

**Table 4:** Leafamine^®^ and water condition effects on salicylic acid, jasmonic acid, abscisic acid and zeatin-o-glucoside ribose content in leaves.

Factor	Endogenous Salicylic Acid (ng/gDW)	Endogenous Jasmonic Acid (ng/gDW)	Endogenous Abscisic Acid (ng/gDW)	Zeatin-o-Glucoside Ribose (ng/gDW)
**Treatment**				
Control	417.4 ± 42.8	87.2 ± 19.8	977.4 ± 502.4	93.2 ± 2.8
	[100]	[100]	[100]	[100]
Leafamine^®^	776.4 ± 152.1	113.0 ± 49.3	2760.8 ± 798.1	143.4 ± 8.8
	[186]	[130]	[282]	[154]
Significance	*	NS	***	***
n	n = 8	n = 8	n = 8	n = 8
**Water condition**				
Well-watered (WW)	492.9 ± 61.5	95.2 ± 20.9	106.3 ± 51.0	101.6 ± 7.4
	[100]	[100]	[100]	[100]
Water deficit (WD)	701.0 ± 167.8	104.9 ± 49.3	3631.9 ± 362.8	135.0 ± 12.1
	[142]	[110]	[3417]	[133]
Significance	NS	NS	***	***
n	n = 8	n = 8	n = 8	n = 8
**Treatment × Water condition**				
Control × WW	421.1 ± 61.0	94.9 ± 28.6	20.4 ± 3.2 c	90.5 ± 1.5 c
	[100]	[100]	[100]	[100]
Leafamine^®^ × WW	612.5 ± 119.6	95.8 ± 45.8	249.5 ± 85.2 c	120.2 ± 15.8 b
	[145]	[101]	[1223]	[133]
Control × WD	411.3 ± 87.0	74.3 ± 36.7	2572.4 ± 196.9 b	97.7 ± 8.0 bc
	[98]	[78]	[12610]	[108]
Leafamine^®^ × WD	874.8 ± 243.3	123.3 ± 82.7	4267.6 ± 238.1 a	157.4 ± 3.6 a
	[208]	[130]	[20920]	[174]
Significance	NS	NS	***	*
n	n = 4	n = 4	n = 4	n = 4

Data are expressed as means ± standard errors of measures taken at harvest (38 days after transplanting), from leaves of lettuces treated and untreated with Leafamine^®^ (0.585g/pot) grown under well-watered and water-deficit conditions. Values were compared by using two-way ANOVA (NS, *, *** non-significant or significant at 0.05 and 0.001, respectively). If Treatment × Water condition interaction was significant, data were subjected to Tukey’s multiple comparison test. Different letters indicate statistical differences for *p* < 0.05. [Base 100 index] is shown for each measurement for each modality.

## Data Availability

Numerical raw data can be provided on request. All other data are presented in the paper.

## Supplementary Materials

The following supporting information can be downloaded at: www.mdpi.com/article/10.3390/ijms23137338/s1.
