# Supplementary material for: Leafamine®, a Free Amino Acid-Rich Biostimulant, Promotes Growth Performance of Deficit-Irrigated Lettuce
Source: Int J Mol Sci. 2022 Jun 30;23(13):7338. doi: 10.3390/ijms23137338 (PMC9266813; doi:10.3390/ijms23137338)
Supplement: Supplementary file 1 [file ijms-23-07338-s001.zip › ijms-1763217-supplementary.pdf]

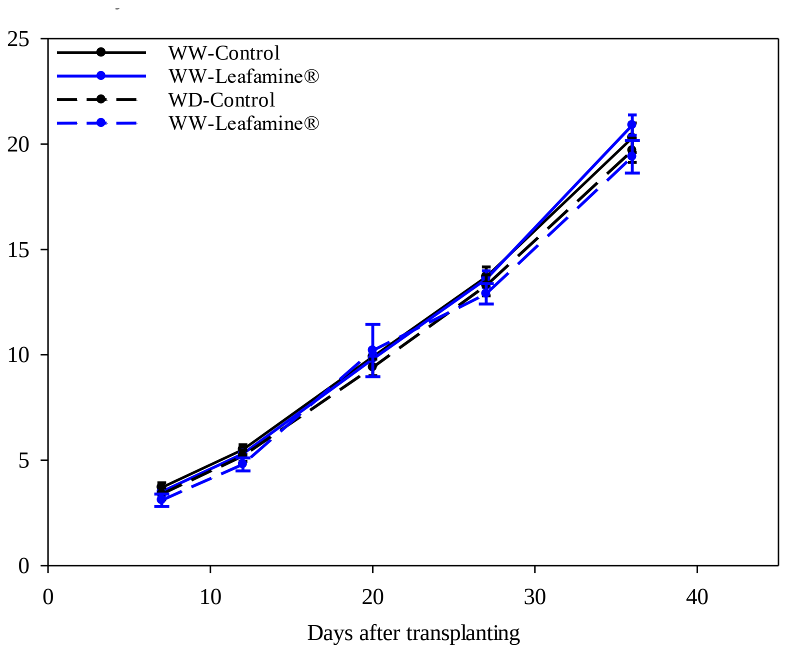

Figure S1. Leafamine® and water condition effects on number of unfolded leaves

Analysis of number of unfolded leaves of lettuces treated and untreated with Leafamine® (0.585g/pot) under well-watered and water deficient conditions. Data are expressed as means ± standard errors of measures taken during the experiment (n=10 lettuces). At each date, values were compared by using two-way ANOVA. Abbreviations: WW-Control, Well-watered lettuces untreated; WW-Leafamine®, Well-watered lettuces treated with Leafamine®; WD-Control, Water-deficient lettuces untreated; WD-Leafamine®, Water-deficient lettuces treated with Leafamine®.

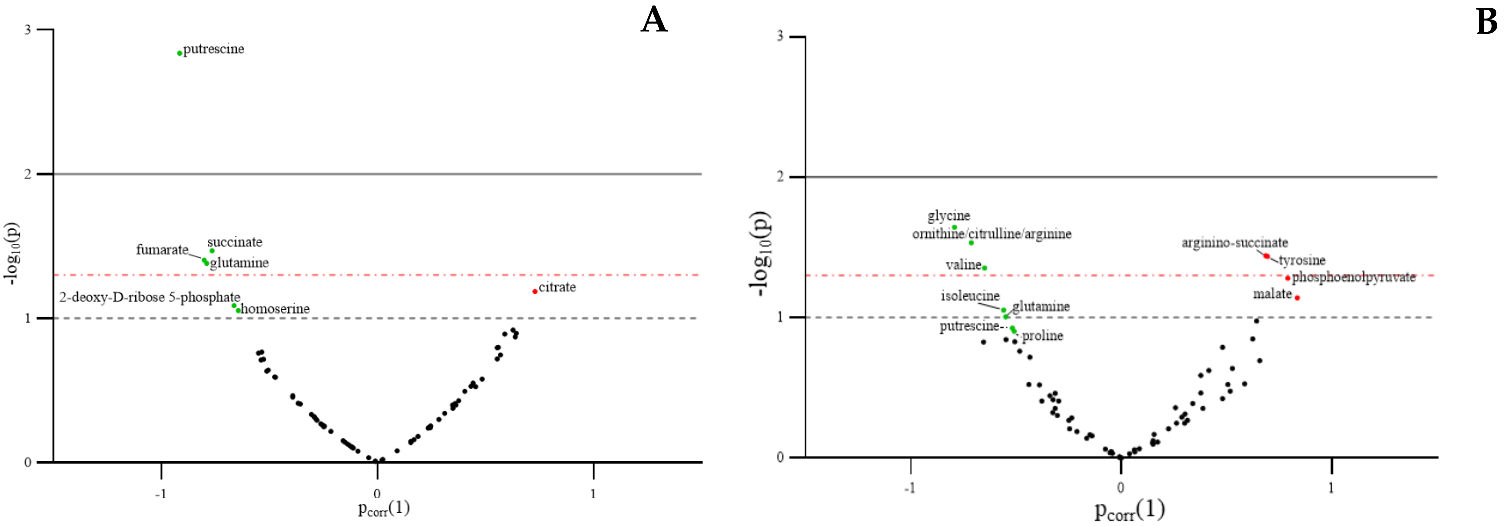

Figure S2. Leafamine® and water condition effects on metabolomics pattern of lettuce leaves 20 days after transplanting

Volcano plot illustrating identified metabolites (Leafamine® application vs control) in lettuces grown under well-watered (A) and water deficient conditions (B) 20 days after transplanting, with the *p*-value (*y*-axis) and the loading in the OPLS-DA (using treatment as ClassID, for each water condition) (*x*-axis). Coloured dots represent differentially expressed metabolites with statistical significance at p-value < 0.1 and 0.05 (overhead horizontal dotted black and red line, respectively) and p-value < 0.01 (overhead horizontal continuous black line). Green and red dots represent metabolites that are up-expressed and down-expressed in Leafamine®-treated and untreated lettuces respectively.

Table S1. Number of lettuces included in each experiment

**Water condition**	**Treatment**	Number of plants **(n)**
Well-watered	Control (water)	10 (trial 1), 12 (trial 2), 35 (trial 3)
Well-watered	Leafamine® - 0,585 g/pot	10 (trial 1), 12 (trial 2), 35 (trial 3)
Water deficient	Control (water)	10 (trial 1), 12 (trial 2), 35 (trial 3)
Water deficient	Leafamine® - 0,585 g/pot	10 (trial 1), 12 (trial 2), 35 (trial 3)

Table S2. General composition of Leafamine®

**Items**	**Results**
Total amino acids *(Method (EC) 152/2009)*	88.9 %
Free amino acids *(Method (EC) 152/2009)*	81.5 %
Na	2.6 %
Cl	2.5 %

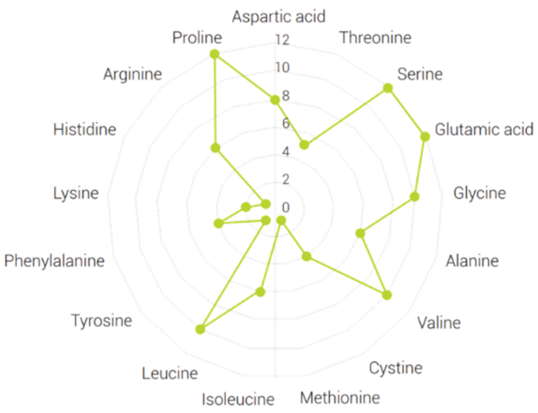

Figure S3. Total Amino Acids profile (in g/100g AA) of Leafamine®

Table S3. Details of Leafamine® composition

Component	Free amino acids	Total amino acids
Aspartic acid	6.7 %	6.8 %
Threonine	4.4 %	4.4 %
Serine	11.3 %	11.3 %
Glutamic acid	9.6 %	10 %
Glycine	7.3 %	7.6 %
Alanine	4.5 %	4.6 %
Valine	5 %	7.5 %
Cystine	1.1 %	1.9 %
Methionine	0.4 %	0.4 %
Isoleucine	3.1 %	4.3 %
Leucine	6.1 %	6.8 %
Tyrosine	0.6 %	0.9 %
Phenylalanine	4.2 %	4.4 %
Lysine	1.5 %	1.7 %
Histidine	0.6 %	0.7 %
Arginine	5.3 %	5.8 %
Proline	9.8 %	9.8 %
**Total**	**81.5 %**	**88.9 %**

Table S4. Composition of peptide fraction (6%) in Leafamine®

**Peptide fraction**	**Results**
> 4 amino acids	7.3 %
3-4 amino acids	31.6 %
2-3 amino acids	61.1 %

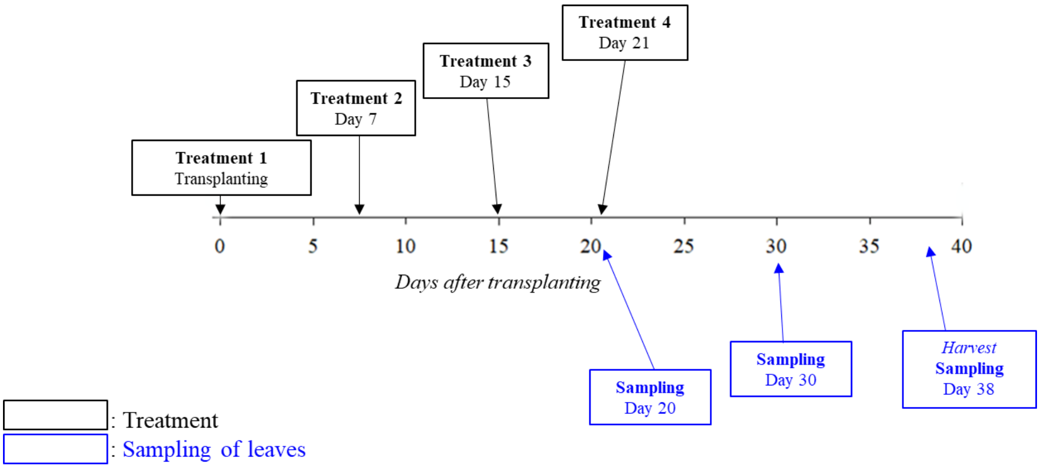

Figure S4. Treatment and sampling protocol of experiment
